# Electronic unified therapy record as a clinical risk management tool in the Italian healthcare system

**DOI:** 10.3389/fpubh.2022.919543

**Published:** 2022-08-03

**Authors:** Giuliano Pesel, Giovanna Ricci, Filippo Gibelli, Ascanio Sirignano

**Affiliations:** ^1^Policlinico Triestino SPA, Trieste, Italy; ^2^Section of Legal Medicine, School of Law, University of Camerino, Camerino, Italy

**Keywords:** digitalization, drug chart, electronic medical record, medication error, therapy

## Abstract

Digitization of health records is still struggling to take hold in the Italian healthcare context, where medical records are still largely kept manually on paper. Besides being anachronistic, this practice is particularly critical if applied to the drug chart. Poor handwriting and transcription errors can generate medication errors and thus represent a potential source of adverse events. In the present study, we attempt to test the hypothesis that the application of a computerized medical record model may represent a useful tool for managing clinical risk and medical expenditure. We shall do so through the analysis of the preliminary results of the application of such a model in two private hospitals in Northern Italy. The results, although preliminary, are encouraging. Among the benefits of digitizing drug records, we recorded a greater accuracy and adequacy of prescriptions, a reduction in the overall workload for nurses (no longer required to manually transcribe the list of drugs from one chart to another), as well as an optimization of the management of drug stocks by hospital pharmacies. The results in terms of clinical risk reduction will be monitored through a prospective cohort study that will take place in the coming months.

## Introduction

On the eve of the fourth industrial revolution, seeing the affirmation—including in the healthcare sector—of advanced technologies such as artificial intelligence and robotics, the existence of paper-based drug charts manually filled by healthcare professionals in the hospitals' wards appears to be an anachronism (1). The use of electronic drug prescription systems, in addition to being more consistent with the digitization and technologization that the current healthcare system has been experiencing for years, ensures greater safety in the delivery of care and could represent a valuable clinical risk management tool.

Concerning the U.S. context, the Food and Drug Administration receives more than 100,000 reports per year related to medication errors (2). More than 7 million US patients are affected by medication errors each year (3). The costs of morbidity and mortality related to prescription drug errors are estimated to be $21 billion annually in the United States (4).

The problem is no less important in the European context. On March 22, 2022, a roundtable debate was organized by the European Alliance for Access to Safe Medicines (EAASM) along with the members of the European Collaborative Action on Medication Errors and Traceability (ECAMET).

The discussion, held virtually with the participation of speakers from European institutions, international organizations and health NGOs, focused on how to prevent medication errors in the European territory. During the debate, the impact of medication errors in the genesis of preventable harm to patients was highlighted, with an estimated 50% of medical service-related harm being attributable to medication-related errors (mainly prescribing and monitoring errors). Among the strategies suggested to curb the spread of the phenomenon, one of the main ones was precisely the use and implementation of technological and IT tools. Within the white paper written in preparation for the event (titled “The Urgent Need to Reduce Medication Errors in Hospitals to Prevent Patient and Second Victim Harm”) (5) it is explained how so-called CPOEs (Computerized Provider Entry Systems) can minimize errors related to medication prescription (6, 7). According to a survey conducted by the paper's editors, e-prescribing systems are widespread in Europe, but poorly integrated with the clinical decision-making process, not available in all departments and not always validated by clinical pharmacists.

Regarding the effectiveness of CPOE systems, there are numerous scientific contributions that attempted to precisely define their impact on the quality of medical care. Among the most significant is an overview of systematic reviews on the topic published in 2020 (8). The study showed that the use of CPOE is associated with a significant reduction in medication ordering errors (9–11), in incidence of adverse drug reactions (9, 11, 12), and in intensive care mortality (10). In contrast, no significant benefits of CPOE systems have been documented in terms of absolute mortality (10, 11) and length of hospitalization (10).

These data provide insight into how CPOEs have enormous potential in terms of improving the quality of health care, but how it takes a long way to get to the point of reaping the best benefits. With particular regard to prescription of drugs, a first and significant step forward has been made by adopting the unified paper-based drug chart, so-called because it concentrates all therapeutic prescriptions in a single document, on which several professionals, mainly doctors and nurses, intervene. This tool greatly facilitated the physicians in carrying out the written prescriptions (which therefore replaced the verbal prescriptions) and allowed to avoid the transcription steps between the medical record and the nursing documentation so that nurses could use the same sheet filled in by the doctor to carry out the administration, saving time and reducing the possibility of transcription errors. Therefore, the unified paper-based drug chart allows to keep track on a single document of all the operations carried out on the process, as well as the author of each intervention, dealing with communication problems, the first cause of medication errors (13). However, since the unified paper chart is still paper-based, it is burdened with all the problems and critical issues that this way of compilation entails (first and foremost, the not always adequate intelligibility of the writer's handwriting).

The next stage of evolution of the therapeutic prescription policy is represented by the transition from handwriting to digital writing through computer tools, already in use in some Italian hospitals.

In this sense, the electronic unified therapy record would represent the digitized version of the paper-based drug chart, maintaining the same purposes and prerogatives but being considerably more practical and manageable and involving a greater quality guarantee in the adequacy of therapeutic prescriptions. Such computerization of the drug prescription system accounts for the increasing involvement in the drug dispensing process of the clinical pharmacist, who through electronic monitoring of drug regimens is able to practice adequate surveillance of drug interactions.

Computerization also makes it possible to dispense drugs remotely, another possibility that makes the enormous innovative potential of the new system very clear. It is precisely in the conviction of the need for such innovation that we chose to conduct the present project, which involved the application of a computerized therapeutic prescription system tool in two private hospitals in the Friuli Venezia Giulia region, in north-eastern Italy.

## From paper-based drug charts to electronic therapy records

### Paper-based drug chart: An obsolete tool?

Although more immediate and practical than the interface with computer systems, the compilation of paper-based drug charts has many drawbacks that may lead to medication errors. The adaptation of diagnostic and therapeutic measures to technological progress is, in fact, a deontological duty of the healthcare professional, as enshrined in the Italian Code of Medical Ethics in Article 78 (“Computer Technologies”): “… the physician must promote the use of information and communication technologies of clinical data for the management of the complexity of medicine and for the improvement of individual and collective prevention tools in particular in the face of clinical and scientific findings that document or justify the preferred choice” (14).

The National Coordinating Council for Medication Error Reporting and Prevention (NCC MERP), an independent international body composed of 27 national organizations with the aim of ensuring safe use of medications and increasing awareness of medication errors by promoting strategies to prevent them, provides the following definition of medication error: “A medication error is any preventable event that may cause or lead to inappropriate medication use or patient harm while the medication is in the control of the health care professional, patient, or consumer. Such events may be related to professional practice, health care products, procedures, and systems, including prescribing, order communication, product labeling, packaging, and nomenclature, compounding, dispensing, distribution, administration, education, monitoring, and use” (15).

There are several different classifications of medication errors. One of the most widely used is the one according to which medication errors can be classified into five macro-categories: prescribing errors, transcription errors, dispensing errors, administration errors, and monitoring errors (16). The written compilation of a paper-based drug chart requires a not negligible commitment by both physicians and nurses, especially in terms of time, which can contribute to the occurrence of medication errors belonging to all five categories, above all transcription errors. Transcription errors can be defined as the product of the inadequate transfer of data from one source to the next, such as copying a drug chart from a complete sheet to a new one (17). A particularly delicate phase of the care process, in which transcription errors can more easily occur, is that of the so-called “medication reconciliation”, i.e., the transcription in the medical record of the therapeutic regime assumed by the patient before being admitted to the hospital (18). Inadequate filling and storage of the paper-based drug chart can pose a serious threat to the proper management of this critical phase. In fact, it is not uncommon for therapy sheets to be confusedly wrapped together, at best protected by a thin transparent plastic cover.

Often these documents, which form an integral part of the patient's medical record, are in very poor condition, creased, dirty (even with blood) and often written with poor handwriting, which generates difficulties of interpretation by the various professionals involved in the treatment process, with all the easily predictable medical-legal consequences. Unfortunately, this situation still represents the norm in many healthcare facilities throughout Italy. This method of drafting and storing the drug chart certainly does not seem to align with the expected procedures of managing a document of public interest such as the medical record, representing a likely source of adverse events capable of affecting the quality of the care process.

### Unified electronic therapy record: The present and future of drug prescriptions

In recent years, more and more healthcare facilities are replacing the paper-based drug chart with its electronic version, the unified electronic therapy record, where “unified” indicates that it can be used by several healthcare professionals, mainly physicians and nurses. This innovative tool, complementary to the electronic medical record, fits within a technological innovation and digitalization process, aiming to lead to an increase in the safety of care (19, 20). Adverse events related to therapy during an inpatient stay are common and costly. Most hospitals identify these events through spontaneous reports (incident reporting).

Computerized approaches to identify such errors seem promising and have been studied since the late 1990s, although it is not easy to compare spontaneous error reporting data with “computerized management” data. In some U.S. studies on this topic, electronic prescribing systems of medical therapies in inpatient wards proved efficient in reducing the risk of major adverse events (21) and also raised the “under-reporting” issue, i.e., a number of spontaneous reports (through incident reporting) significantly lower than reality (22). During the 2000s, even in Italy, experiments with electronic prescribing systems began. They become particularly widespread in recent years thanks to the spread of Wi-Fi networks and, in general, computer systems' progress. In the following years, a heated debate also began in the scientific community, primarily American but also British, about the effectiveness or, on the contrary, the dangerousness of electronic prescription systems. Computerized drug prescribing has long been touted as a significant improvement in patient safety, primarily due to the 1999 American Institute of Medicine report on errors (23).

Although the literature suggests that such systems can improve patient outcomes through decreases in adverse drug events, actual improvements in medical outcomes have not been documented. In fact, according to some authors, the implementation of such systems may increase the number of adverse drug events and result in higher overall medical costs, particularly in the early years of their adoption, which is undoubtedly the downside (24).

## The healthcare context within which the project was developed

### Clinical risk management and incident reporting in the friuli venezia giulia region

Incident Reporting is a system that allows to detect situations of risk to the safety of operators and users due to critical organizational issues and errors (25). This tool allows to report and describe situations that can potentially result in adverse events (i.e., patients' health problems more likely attributable to treatment errors than to the underlying pathologies) or in near-miss events (i.e., situations in which patients are exposed to potentially dangerous conditions but in which harm does not materialize due to accidental circumstances or implementation of adequate protective measures) (26). The main purpose of this voluntary and anonymous reporting system is to develop a culture of non-guilt on the part of the operator who makes a mistake or reports an error or non-compliance with the culture of safety. The system should raise awareness of risk perception, detection, and management. The possibility of “learning from experience” must be seen as an opportunity to avoid repeating reported events and improve the continuous cycle of safety and quality of care. The collection and analysis of adverse events and events avoided is an essential pool of data and information for the mapping of areas at higher risk at a corporate level. The subsequent analysis of an adverse event or an event avoided is essential to increase awareness of the organization's level of safety and acquire critical information for the management of clinical risk and improvement actions to be taken.

From the clinical risk surveillance activity carried out at the Policlinico Triestino by the Health Departments through the analysis of incident reporting, it has emerged that reports related to the incorrect prescription/administration of drugs represent a significant proportion of all reports. Among these, the use of paper-based drug charts represents a considerable share. The “Safe Care Network”, established by regional resolution of the autonomous region of Friuli-Venezia-Giulia No. 1970 of October 21, 2016 (27), coordinates and governs, in an integrated form, the activities related to the safety of care. In addition, through Regional Resolution No. 185 of February 2, 2018 (28), it fulfills the functions of “Center for Health Risk Management and Patient Safety” according to the requirements of Law No. 24 of March 8, 2017 (the latest reform of healthcare liability and safety of care legislation in Italy). The “Safe Care Network” coordinates and governs, in an integrated form, the activities related to the safety of care. Participation is mandatory for all entities belonging to the National Health Service and for private hospitals affiliated with the Regional Health Service. Policlinico Triestino actively participates in the network. The activities carried out by the network, launched in 2010 with the “Clinical governance and patient safety in Friuli Venezia Giulia” program, were officially defined with Resolution No. 1970 of October 21, 2016. The Network consists of the central directorate for health, social policies and disability, the regional coordinating agency for health, the corporate risk managers, the corporate managers of regional programs, the corporate link professionals, the healthcare professionals belonging to the Regional Health System, the citizens.

The program consists of several projects developed to ensure adequate standards of safety and quality shared between hospital and territorial context:

1. Safe use of medications
2. Prevention and control of care-related infections
3. Prudent use of antibiotics (antimicrobial stewardship)
4. Safety of clinical care practices
5. Citizen involvement
6. Prevention of violence against providers

### Policlinico triestino: The birthplace of our project

Policlinico Triestino is the most important private healthcare facility in the Friuli-Venezia Giulia Region (North-Eastern Italy). It includes two private hospitals affiliated with the National Health Service in the Province of Trieste and ten medical clinics with a blood drawing center scattered between Trieste and Gorizia and their provincial territories. Concerning the two private hospitals, the first, “Salus”, provides outpatient and inpatient healthcare services for medical, surgical and specialist pathologies, has over 400 hospital admissions per year (consisting of 74 beds). It has a testing laboratory that analyses ~1,000,000 blood samples per year and provides about 60,000 radiological and outpatient services. The second one, “Pineta del Carso”, can rely on 70 beds and is divided into a neuro-motor rehabilitation ward, a respiratory rehabilitation ward, a hospice ward and a severely disabled ward (intended for patients with severe central nervous system injuries). It is focused mainly on rehabilitation-type treatments.

Considering the 2 private hospitals and 10 medical clinics, services related to almost all medical and surgical specializations are provided in the private regime and in convention with the National Health System. All healthcare facilities accredited with the National Health System in the Friuli Venezia Giulia Region and the other Italian regions undergo thorough periodic audits to verify the existence of the conditions of eligibility for renewal of accreditation. These audits include the verification of numerous items related to the quality and appropriateness of care. One of the most essential aspects assessed in institutional accreditation with the National Health System in the Friuli Venezia Giulia Region is the monitoring of incident reporting, i.e., the reporting of adverse events related to healthcare, including those resulting from incorrect prescription or administration of drugs.

Policlinico Triestino has undertaken for many years a process of digitalization of the medical record through a program provided by a software-house of national importance.

The latest step in the ongoing innovation is the transformation of the traditional paper-based drug chart into an electronic version (unified electronic therapy record), which is being tested in some wards of one of the two private hospitals and whose use will then be extended to all the facilities of the group. The need underlying this process is the rationalization and speeding up of care processes and the reduction of clinical risk arising from the administration of drugs. There are also numerous advantages in terms of organization and economics, particularly regarding the pharmacy warehouse. In fact, the computerization of the prescription and administration of medical therapy also allows for better management of stocks in the warehouse.

### Incident reporting results related to medication errors over the 2015–2020 period

From the constant monitoring activity by the Health Departments involved in this study, 11 reports were recorded from “Salus” private hospital and 14 from “Pineta del Carso” private hospital.

These reports refer to criticalities linked to the use of the paper-based drug chart in the period between 2015 and 2020. Regarding the outcomes, it should be noted that these were primarily near-miss events with early detection and immediate correction or events with no outcome or negligible outcome.

In very few cases there were minor outcomes. However, it is likely that, in the absence of active surveillance of adverse events, the events recorded could also have caused significant consequences on patients.

Within Table 1, the spontaneous reports related to the use of the paper-based drug chart in the period between 2015 and 2020 in the two private hospitals afferent to the Policlinico Triestino are detailed. Level 3 events are those in which there is no harmful outcome to the patient, while Level 4 events are those in which there is a minor, negligible outcome not requiring specific treatment. It should be noted that the “Pineta del Carso” private hospitals became part of Policlinico Triestino only in November 2019. The number of reports from the “Pineta del Carso” private hospital results higher, while the “Salus” private hospital shows a higher severity index (more level 4 events). This is a predictable result, since the “Salus” private hospital treats patients suffering from acute conditions, while the “Pineta del Carso” private hospital mainly treats patients suffering from chronic illnesses.

**Table 1:** Spontaneous reports related to the paper-based drug chart in the period 2015–2020 in the two private hospitals of Policlinico Triestino.

	**“Salus” nursing home**	**“Pineta del Carso” nursing home**
Near-miss events	2	3
Level 3 events	5	10
Level 4 events	4	1
Total	**11**	**14**

## The unified electronic therapy record adopted in the context of the present study

### The features of our IT tool

Given the company's need to switch to an IT tool for therapy management, this program was chosen because it complements the medical records management system already in use at Policlinico Triestino and is supplied by the same software house that has supplied similar systems in use in other Italian hospitals. The application is based on a server platform that is regularly backed up and guarantees data availability. Passwords and sensitive data are protected and are not easily accessible. The separation of data is guaranteed, and the system respects the current legislation on privacy. Referential integrity functions present in all versions of Oracle Databases are activated in the database (referential integrity is a software property that ensures that relationships between different tables are consistent; the Oracle database is one of the most popular database management system software). There is also a module designed to guide the preparation and administration phases of therapies that require dilution by the nurse. The patient dashboard module (dashboard) is the main screen of the program. It immediately highlights the status of patients and clinically relevant activities that are required or scheduled and allows access to further modules. The patient dashboard module has a set of configurable filters that allow to search for patients more quickly. Additional filters can be applied to the subset that identifies “Inpatient Department” patients (admitted and bedridden in the user's department) to select patients based on recorded data or assigned beds. For each selected patient, the relevant personal and clinical information is reported (name, tax code, sex, date of birth, age, hospitalization, bed identification, etc.). For each anagraphic position, allergies/intolerances or other relevant notes recorded in the medical record can be retrieved (configurable according to the specifications provided by the client). This information is configurable depending on the specifications provided by the client (clinic or hospital).

The prescription management module provides for the recording and confirmation of the following information:

- Identifier of the prescribed drug
- Route of administration
- Dosage and duration of administration for “continuous” prescriptions (e.g., intravenous, transdermal, etc.)
- Type of prescription (at exact times; at time slots; as needed)
- Frequency of repetition (daily; every other day; on a schedule; etc.)
- Prescription start and end date
- Type of therapy (chronic/acute)

**Figure 1:**
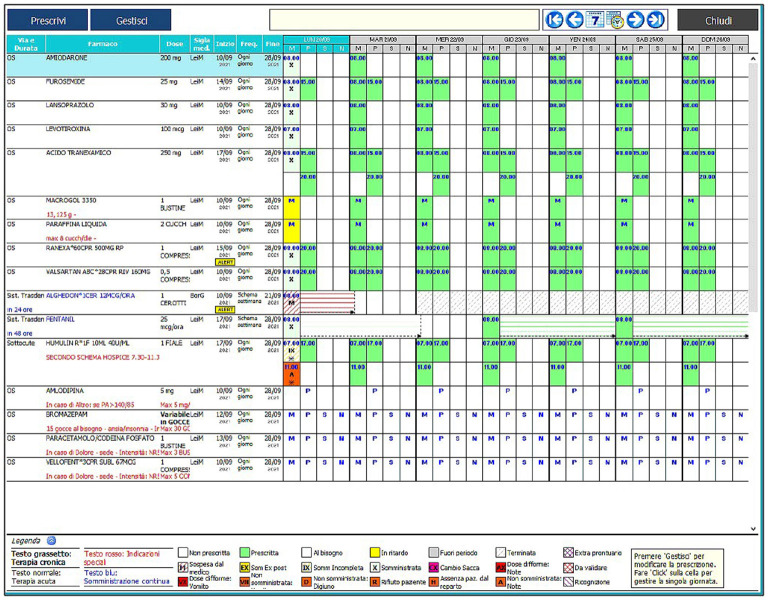
The main screen of the unified electronic therapy record employed in the present study.

Numerous alerts allow to monitor various aspects of the prescription/drug administration process. The physician's functions cannot be managed by the nurse and vice versa, and the program is set up to have a clear consequentiality between the actions of the two professionals. All these features make the program a completely reliable and safe tool. Figure 1 illustrates the main screen of the computer program.

The system has 5 basic features:

1. Modularity and customizability: it maintains an incomparable consistency and compactness, adapting to specific clinical and organizational needs.
2. Transactionalness: it manages the granular control of each phase of the process and builds data collections for the control and monitoring of the quality and quantity of the activities carried out, also with the evaluation of costs and results.
3. Completeness and structuring: it manages the completeness of the data, supporting the medical-nursing activity through the management of care protocols and the standardization of information.
4. Openness and transparency: it easily communicates with different equipment and systems, increasing the quantity and quality of immediately available information, for maximum interoperability and reduction of delays and omissions. The system supports the International standards for communication in the health sector (DICOM, HL7, IHE): the integration of the various information systems already present at the customer will take place with standard tools and protocols.
  In detail, the system complies with several open standards:

○ HL7/IHE/Web service for connectivity with systems;
○ HTML5 for viewing on all browsers;
○ Standard SQL DBMS: The Oracle database is a standard and can be queried *via* SQL code in standard SQL-99 core, using Open Source tools, such as SqlTools (available on Sourceforge.net), with the possibility of extracting files in standard CVS format.
  Adherence to these standards allows you to connect the system offered to any other system using open standard formats and to be able to extract data even without resorting to proprietary tools.

5. Flexibility and scalability: it easily adapts to the evolution of processes, new technologies and changes in organizational models, even expanding on a large scale toward territorial solutions.

### Operational aspects

The unified electronic therapy record object of this study shows, for each selected patient, unequivocally, the name of the drug and its dosage, the administration route (each marked with a different color card), the days on which it is to be administered, the time and any notes in the notes box (e.g., dilutions). The system informs the doctor during the prescription phase if the drug he is about to insert is already part of the treatment plan, it shows all the drugs on the market for the same active ingredient, being connected to the program. Whether doctor or nurse, each user enters the program after typing in an identification code and a password so that any operation carried out by him, both on the hardware and the software, is traced. Each user has his own operating profile in relation to the role he performs, so a doctor can generally prescribe, modify, and eliminate a therapy. A nurse can administer it but cannot complete the functions attributed to the doctor. It is possible to view the history of prescribed and administered therapies at any time, distinguishing the operator, the day, date, and time when the operation was carried out. This documentation can be printed when the patient is discharged and attached to the medical record. The software also allows the patient's prescription to be filled without re-entering personal data and having the complete sheet of all the therapy. The system can automatically generate periodic and urgent requests for the restoration of pharmaceutical products needed in the warehouse, taking into account the actual need, the values of the minimum limit (the stock value below which the system generates an order), and the reorder threshold (the value to which the system reports the stock of the drug each time a restoration order is generated) based on specific calculations that take into account the individual doses prescribed.

In our opinion, a unified electronic therapy record provided with the features listed can effectively address three of the five categories of medication errors listed above. Concerning the errors most frequently attributable to the manual drafting of the drug chart, the transcription errors, these are substantially annulled since the doctor prescribes the therapy using a computer (desktop computer or portable computer) and the nurse uses both the same source of data and the same technological support for the administration. This implies, on the one hand, time savings for the nursing staff, who no longer have to transcribe from the medical record into special registers or notebooks used for administration, and on the other hand a reduction of reading errors, since the therapies are clearer and more straightforward to read and interpret as they are written through a computer system. The unified electronic therapy record is also able to reduce the incidence of prescribing and dispensing errors. As far as prescription errors are concerned, the computer system is beneficial since it can provide complete information about the therapy (the correct name of the patient, name of the drug, dosage, time of administration, etc.) and since it prevents the prescription from proceeding until all the fields have been entered. It also helps the doctor to restrict the choice of drugs to those belonging to the formulary or available in the hospital. A link to Federfarma's database is even available to clarify any doubts about the active ingredient or commercial name (Federfarma is the national federation of Italian pharmacy owners).

The drug register can be automatically aligned and currently updated to the Federfarma database (National Federation of Italian pharmacy owners), with the possibility of filtering the categories of drugs to be treated. Through a dedicated tool, all the periodical updates of the FEDERFARMA database are loaded directly into the Oracle database on coding tables that contain both active ingredients and commercial drugs. Each active ingredient is identified by the unique identifier code of FEDERFARMA (codpa); each commercial drug is identified by the AIC code (identification code for medicinal products for human use). So, in the prescription modules, when choosing or consulting drugs and therapies, it is always possible to access the detailed sheet showing, in addition to the MINSAN and EMEA code, the following information sections:

➢ PRODUCT DATA
➢ COMPANY AND SALES DATA
➢ MONOGRAPHY
➢ INTERACTIONS
➢ EQUIVALENT DRUGS

About the administration of drugs, the electronic system does not provide an absolute guarantee of the univocity between the patient and the correct medication to be administered since, by our choice, the electronic reading of the patient's bracelet barcode and the AIC code (identification code for medicinal products for human use) of the drug is not active even though it is predisposed to this operation. To alleviate the difficulties of the change, we decided to postpone the operation to a later stage, gradually getting staff used to the new operating system. Furthermore, the system helps to reduce the likelihood of administering the drug by a different route than the prescribed one, it allows the printing of therapies by selecting the route of administration, it warns the operator if he is administering a drug before the scheduled time, it doesn't allow the administration of a drug outside the scheduled day (or if it had been suspended) and it notifies any therapies to be administered or delayed.

### Economic aspects

Another relevant aspect of the problem arising from the correct prescription and administration of drugs during the treatment process is undoubtedly economic. The process of drug management can be improved through different technical choices, such as computerization of operations, computerized cabinets, unit dose distribution, and other forms of customization. Depending on the technical choice made, different degrees of improvement in the entire process are achieved. In the first phase, the immediate activation of the “warehouse-pharmacy management” module is not foreseen. Still, as soon as the healthcare staff is ready (i.e., adequately trained) this module will also be activated, given the proper importance of this aspect. The use of the new IT package in full mode began in late 2021.

In fact, an appropriate use of the drug is crucial to improve the patients' health status and optimize the allocation of economic resources. It is essential to experiment with information technologies that support operators along the whole path from prescription to administration of drugs, to the verification of the administration, up to the traceability in the levels of responsibility. The computerized therapy prescription by doctors is unambiguous, without further transcription, resulting in fewer errors and further levels of control between the prescription and preparation. Nurses can thus save time that they can spend to care for patients, as it is free from repetitive activities that the traditional system imposes, focusing exclusively on administration. On the economic side, the system effectively reduces the consumption of drugs and stocks, entailing a radical change in the organization that requires an articulated training of all actors involved: pharmacists, physicians, and nurses. With the computerized system, stocks and incoming and outgoing flows can be recorded. The system will then provide as a final step the reading of the bar code of the patient's wristband with the AIC code of the drug packages to confirm the correct assignment of the therapy.

### Preliminary results

The innovation project based on the unified electronic therapy record application saw the end of experimentation for both private hospitals in March 2022, with the final transition to the new system in all departments. In the “Pineta del Carso” private hospital, where experimentation has begun, the new system became fully operational in late 2021. In the meantime, the new system will be monitored and studied both from the point of view of clinical risk and from the perspective of the management of pharmacy warehouse resources by the pharmacist and the purchasing office. The Policlinico management is also studying the proposal to administer to all personnel involved an anonymous questionnaire to detect the satisfaction with the new IT tool, in two different moments, as at the beginning and end of the experimentation.

The main issue we foresee is the increased risk of errors in the transition phase between the use of the old system and the new system. In recent months, a staff awareness program is being implemented, alongside training in the use of the computer program, regarding the need to report errors. An increase in active error surveillance by risk managers assisted by nurse coordinators is essential in this phase.

In terms of feedback from healthcare professionals, we found a greater tendency for younger nurses and physicians to welcome the innovation, the older ones being more reluctant to use IT tools.

### Medico-legal aspects: The nurse's role

In recent years, nurses have assumed an increasingly central role in the therapeutic process offered to patients, mainly as they possess a more and more rich and deep medical background. From the mere task of administering the drug upon prescription (conception of the logic of “job”) currently, the nurse is the guarantor of the correct application of diagnostic and therapeutic prescriptions. Within the therapy process, the nurse is required to play a role of “feedback”, with a view to a collaborative vision with the physician, but at the same time an antithetical role when the need to protect the patient arises. At this level, an IT tool such as the unified electronic therapy record can provide valuable help. The problem of deaths from medication errors (which are legally qualified as “foreseeable and avoidable” by jurisprudence) has long required risk reduction strategies that involve the therapy process in the totality of its phases: procurement, storage, prescribing, preparation, distribution, administration, and control. In this process, the nursing responsibility finds its first source in the professional guidelines. The postulates of the correctness of action reside in the following rules: correctness of the drug and the dose, correct identification of the patient, right way and time of administration, registration, control. Errors during the various stages of the therapeutic process fall on the nurse in the first instance. In the event of damage caused to the patient, elements of civil (compensatory) and criminal liability may arise.

The jurisprudence (29, 30) emphasized, as a result of the limits of the principle of reliance (corresponding to obvious factual situations that reasonably cast doubt on the occurrence of compliance with the duties of diligence, skill and prudence, by their collaborators), that the nurse must detect obvious inappropriateness of therapeutic prescriptions, in particular for gross errors in the indication of the dosage, posology or prescription of drugs to which the patient is allergic and then report them to the doctor for appropriate revisions. With Judgment No. 1878 of October 25, 2000, the Supreme Court of Cassation declared guilty of manslaughter a physician and a nurse for causing the death of two patients following the administration of an inappropriate dose of potassium chloride. Specifically, the medication initially supposed to be administered (potassium chloride) had been replaced by a similar solution but containing a different potassium concentration. Upon learning of the fact, the ward physician had merely given generic and superficial verbal instructions to the nurse who was about to administer the drug materially. The nurse did nothing to induce the doctor to modify the prescription (recalibrating the dosage of the solution) and proceeded to administer the deadly drug. The judges held that: “… in case of doubts about the prescribed dosage, the nurse must take action not to syndicate the therapeutic efficacy of the prescribed drug, but to draw attention to it and request the written prescription renewal”. According to the Supreme Court, the nurse has “… a specific duty to attend the activity of drug administration in a non-mechanistic way (i.e., measured on the level of an elementary fulfillment of tasks merely executive), it is necessary instead to intend the performance in a manner consistent with a form of collaboration with medical staff oriented in critical terms …”.

On January 16, 2015, with Judgment No. 2192, the Court of Cassation declared guilty of manslaughter a nurse who, despite being aware of it, had not reported to the doctor an error in the prescription of a drug to a patient allergic to a substance contained therein. The judges reasoned the decision as follows: “… in consideration of the quality and the corresponding content of the relevant professional activity, it is impossible not to recognize the existence, for the nurse, of a precise duty to attend to the activity of drug administration in a non-mechanistic way (i.e., measured on the level of an elementary fulfillment of merely executive tasks); on the contrary, it is necessary to intend its fulfillment according to modalities consistent with a form of collaboration with the medical staff oriented in critical terms; and so much, not already in order to syndicate the work of the doctor (particularly in terms of the therapeutic efficacy of the drugs prescribed), but in order to draw attention to the errors perceived (or otherwise perceivable), or in order to share any doubts about the adequacy or relevance of the therapy established with respect to the hypothesis subject to examination, from these premises resulting in the use of timely legal obligations to activate and solicit time to time specifically and objectively determinable in relation to each concrete case …”. The responsibility landscape dictated by the Supreme Court exposes the nurse to a delicate role of verification (in addition to the known panorama of contraindications and post-recruitment events) that is contiguous to the task of translation of what the doctor prescribes (not transgressing the canons of risk management). This results from the fact that the team is horizontal and involves active collaboration between physician and nurse. Instead, there is a real obligation of control, immune from the principle of tempered trust, in the case of delegation of the act of administering oral therapy to support figures (social-health professionals) that requires the nurse (by virtue of the verticality of the performance of the team considered) to verify the correctness of the work of others (in this case the accuracy of the route of administration, the mode of administration and the recruitment).

## Conclusions

To date, the innovation project is in the starting phase at the “Pineta del Carso” private hospital, and the first results after 1 month of experimentation are encouraging. There has been no increase in spontaneous reports of therapy-related errors. These preliminary results are interesting, but it is likely that the change is succeeding because the attention of the operators is, at the moment, very high on the subject (also due to pressure from the company management). It still takes time to get a complete perspective.

At the end of the experience of the introduction of the electronic unified therapy record, the nursing staff and the physicians of lower age and seniority proved to be enthusiastic about the change and very cooperative, while the same cannot be said for the “older” staff, traditionally more reluctant to change and to the use of IT tools. The results in terms of clinical risk reduction will be monitored through a prospective cohort study that will start in the coming months, after the adoption of the IT system in all departments. The study will use incident reporting to measure the effectiveness of the innovation system. Preliminary results will be available in late 2022. Initial economic data related to the revamped computerized medication and inventory management will be available simultaneously.

## Data availability statement

The original contributions presented in the study are included in the article/supplementary material, further inquiries can be directed to the corresponding author.

## Author contributions

GP came up with the original idea and followed the developments of the application of the IT system in the two nursing homes. FG wrote the draft article with the support of GR and AS. GR and AS supervised the project. All authors contributed to the article and approved the submitted version.

## Conflict of interest

Author GP was employed by Policlinico Triestino SPA. The remaining authors declare that the research was conducted in the absence of any commercial or financial relationships that could be construed as a potential conflict of interest.

## Publisher's note

All claims expressed in this article are solely those of the authors and do not necessarily represent those of their affiliated organizations, or those of the publisher, the editors and the reviewers. Any product that may be evaluated in this article, or claim that may be made by its manufacturer, is not guaranteed or endorsed by the publisher.
